# Importance of glycated hemoglobin in hyperglycemia diagnosis of patients with sepsis

**DOI:** 10.1186/cc10163

**Published:** 2011-06-22

**Authors:** ACM Simioni

**Affiliations:** 1 EPM-UNIFESP, São Paulo - SP, Brazil

## Introduction

Hyperglycemia is a frequent event in patients hospitalized in intensive care units (ICUs) and was attributed to endocrine metabolic stress related to acute disease. However, the interference of diabetes mellitus (DM) or undiagnosed glucose intolerance in hyperglycemia pathogenesis in critically ill patients is not well established.

## Objective

To correlate the presence of DM with hyperglycemia or glucose intolerance, not previously diagnosed in patients with severe sepsis/septic shock in the ICU, using the new standards of the American Diabetes Association (ADA) for classification of glycated hemoglobin (HbA1c) [1].

## Methods

A study prospectively evaluating patients admitted to the ICU between the January 2007 and August 2009. We included patients with severe sepsis or septic shock, with less than 48 hours from organ dysfunction onset. Severe sepsis and septic shock were defined based on International Sepsis Definitions Conference criteria [2]. Exclusion criteria were: previous diagnosis of DM, insulin infusion at the time of evaluation, sepsis within <30 days and refusal to participate. According to new ADA classification, patients were considered normal with HbA1c ≤5.6%, glucose intolerant with HbA1c between 5.7% and 6.4% and diabetic those with HbA1c≥ 6.5% [1]. Statistical analysis used the *t *test, chi-square and correlation coefficient and was made using SPSS 15.0 software.

## Results

**Figure 1:**
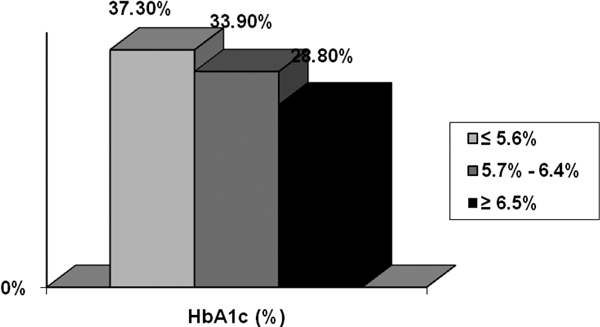
**Presence of diabetes or glucose intolerance undiagnosed**.

Our sample included 59 patients, mean age 60 ± 18 years, 62.7% were male. By classifying patients according to HbA1c, although denying a history of DM, only 37.3% had normal HbA1c. About 28.8% had undiagnosed diabetes and 33.9% had glucose intolerance. Analyzing the HbA1c as a continuous variable, we found only a statistically significant correlation with blood glucose levels at inclusion (*P *= 0.04), serum insulin at inclusion (*P *= 0.02) and insulin resistance at inclusion (*P *= 0.02). Studying the population characteristics, an association between HbA1c change and presence of comorbidities was observed (*P *= 0.004). Furthermore, patients with HbA1c changes were older (*P *= 0.02), had higher blood glucose at inclusion (*P *= 0.03) and higher lactate after 24 hours of inclusion (*P *= 0.03). See Figure 1.

## Conclusion

In this sample of patients with sepsis without previous history of DM a high incidence of patients with diabetes and glucose intolerance undiagnosed was found. Therefore, HbA1c measurement in the ICU may be useful in the investigation of patients with hyperglycemia.
